# The Nursery School

**Published:** 1920-01-31

**Authors:** 


### THE NURSERY SCHOOL.

MISS MARGARET MCMILLAN has just written a book on *The Nursery School*, which is of interest to all who are in any way connected with child welfare. She describes an open-air nursery school in the heart of Deptford, and gives a great deal of valuable information on matters of finance, buildings, and method. The school, according to a review in the *Manchester Guardian*, is turning out a wonderful new generation of healthy "nurtured" children, whose advent into the ordinary council schools at the age of seven is going to necessitate the entire overhauling of those places. The children, from one to seven years old, are housed in a series of shelters whose sides are made of movable screens, so that they can be practically out of doors all the time, though protected from wet and wind. Much care is taken with the physical training of the little people, who are taught very early to wash and dress themselves and wait on each other at table. They wear simple but charming clothes, and their sense of beauty is drawn out through sight, sound, and touch.

Physically, mentally, and morally the results in these slum children are wonderful, and for those who are doubtful of the benefits of the fresh air found in thickly populated districts in winter it may be interesting to note that the best health records in an open-air nursery are in January. Students who attend the college, which is run in connection with the school, as "teacher-nurses" have training in the dental and medical clinics attached to the centre and attend lectures in psychology, handwork, music, and art, as well as doing practical work in the nursery. They should become well equipped.

